# Environmental Surveillance as a Tool for Identifying High-risk Settings for Typhoid Transmission

**DOI:** 10.1093/cid/ciaa513

**Published:** 2020-07-29

**Authors:** Jason R Andrews, Alexander T Yu, Senjuti Saha, Jivan Shakya, Kristen Aiemjoy, Lily Horng, Farah Qamar, Denise Garrett, Stephen Baker, Samir Saha, Stephen P Luby

**Affiliations:** 1 Division of Infectious Diseases and Geographic Medicine, Stanford University School of Medicine, Stanford, California, USA; 2 Child Health Research Foundation, Department of Microbiology, Dhaka Shishu Hospital, Dhaka, Bangladesh; 3 Dhulikhel Hospital, Kathmandu University Hospital, Dhulikhel, Nepal; 4 Department of Pediatrics and Child Health, Aga Khan University Hospital Karachi, Karachi, Pakistan; 5 Sabin Vaccine Institute, Washington, DC, USA; 6 Department of Medicine, Cambridge Institute of Therapeutic Immunology & Infectious Disease (CITIID) University of Cambridge, Cambridge, United Kingdom

**Keywords:** typhoid, enteric fever, *Salmonella*, water, environment

## Abstract

Enteric fever remains a major cause of morbidity in developing countries with poor sanitation conditions that enable fecal contamination of water distribution systems. Historical evidence has shown that contamination of water systems used for household consumption or agriculture are key transmission routes for *Salmonella* Typhi and *Salmonella* Paratyphi A. The World Health Organization now recommends that typhoid conjugate vaccines (TCV) be used in settings with high typhoid incidence; consequently, governments face a challenge regarding how to prioritize typhoid against other emerging diseases. A key issue is the lack of typhoid burden data in many low- and middle-income countries where TCV could be deployed. Here we present an argument for utilizing environmental sampling for the surveillance of enteric fever organisms to provide data on community-level typhoid risk. Such an approach could complement traditional blood culture-based surveillance or even replace it in settings where population-based clinical surveillance is not feasible. We review historical studies characterizing the transmission of enteric fever organisms through sewage and water, discuss recent advances in the molecular detection of typhoidal *Salmonella* in the environment, and outline challenges and knowledge gaps that need to be addressed to establish environmental sampling as a tool for generating actionable data that can inform public health responses to enteric fever.

Enteric fever, a systemic bacterial infection caused by *Salmonella enterica* subspecies *enterica* serovars Typhi and Paratyphi A, B, and C, remains a significant cause of morbidity and mortality globally. It is estimated that 12–16 million new cases of enteric fever arise each year, which result in 77 000–219 000 deaths. The overwhelming majority of the disease burden occurs in low- and middle-income countries (LMICs) in South Asia, Southeast Asia, and sub-Saharan Africa [1]. North America and Europe largely eliminated enteric fever through the provision of clean municipal water and sanitation measures in the early 20th century; however, similar large-scale civil engineering projects have not been sufficiently transposed to resource-constrained communities in LMICs where typhoid remains endemic [2].

Vaccines against typhoid have been available since the 1890s, but their efficacy, safety profile, and the durability of the protective immune responses have limited their suitability for national immunization programs. Consequently, very few LMICs have incorporated typhoid vaccines into their routine immunization programs at national or even subnational level. More recently, TCVs have demonstrated greater efficacy and more durable immune responses than previous generations of typhoid vaccines [3], leading the World Health Organization to issue a new recommendation that TCVs be utilized in countries with a high incidence of typhoid or with a high degree of antimicrobial resistant *S*. Typhi [4]. The Global Alliance for Vaccines and Immunisation (Gavi), the Vaccine Alliance (Gavi), has agreed to support the introduction of TCV into LMICs, many of which are assessing how to prioritize vaccination against typhoid against vaccine-preventable diseases not currently covered by their national immunization programs.

A major difficulty for countries facing decisions about whether to introduce TCVs into their national immunization programs is the scarcity of data on the burden of enteric fever. Enteric fever is difficult to distinguish clinically from other acute febrile illness, and available diagnostics perform poorly [5]. Blood culture remains the only real definitive approach to diagnose enteric fever; however, the laboratory capacity for blood culture is typically limited in communities where typhoid may be endemic. Additionally, it is common that individuals with acute febrile disease may seek care at pharmacies or private medical providers where a blood culture is not performed. Few LMICs have a sustainable microbiological surveillance system for enteric fever, and even fewer have nationally representative data on the burden of enteric fever. Some LMICs do have incidence data generated via short-term population-based research studies, yet these data are frequently geographically and temporally limited. The majority of Gavi-eligible countries that may have endemic enteric fever lack the basic essential data to inform critical decisions for TCV introduction, as well as systems for monitoring ongoing typhoid burden and vaccine impact.

Given the current lack of enteric fever burden data there is clearly a need for affordable and sustainable enteric fever surveillance methods for that could be scaled in LMICs (Figure 1). “Hybrid” surveillance models, which combine facility-based case detection with community-based healthcare utilization surveys, are one potential strategy for containing costs while generating reasonable population-based incidence estimates [6]. However, this approach requires blood culture capacity and human resources for the planning, execution, and analysis of large, prospective surveys. Although this hybrid approach is now being used in studies in South Asia and sub-Saharan Africa, to date, no national surveillance programs have used their own resources to implement such an approach. Sero-epidemiology, a measurement of antibody responses against enteric fever organisms through population-based surveys, has yielded some promising results [7], but the optimal antigen/antibody combinations and the relationship between immune responses and enteric fever incidence requires clarification.

**Figure 1.**
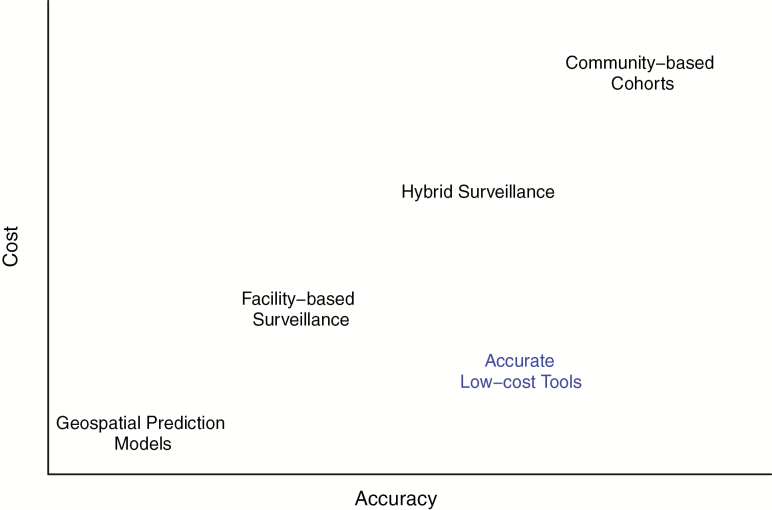
Accuracy and cost tradeoffs in enteric fever burden estimation. Currently used modalities for enteric fever surveillance range from geostatistical prediction models, which draw upon data from other times and places to make estimates in places where contemporary primary data are not available, to prospective, population-based cohort studies, which are costly but can generate direct population-based estimates of disease incidence. Facility-based and hybrid facility- and community-based surveillance fall between these extremes in terms of cost and accuracy. There is an urgent need for new, low-cost approaches that achieve reasonable accuracy in estimating enteric fever disease incidence. Environmental surveillance represents one such candidate.

An emerging approach is the application of environmental surveillance methods for detecting the circulation of *S.* Typhi and *S*. Paratyphi A. Environmental surveillance encompasses collecting samples from matrices involved in pathogen transmission, such as drinking water, sewage, air, or fomites, and screening them for evidence of pathogens or other indicators of microbial contamination. Such testing can be performed prospectively to detect pathogens prior to the recording of clinical cases or to monitor their abundance in environment to assess the potential risk of disease. Alternatively, environmental surveillance may be conducted reactively after the identification of an outbreak to assess the potential magnitude and identify the source. Examples of environmental surveillance for other (nonenteric fever) infectious diseases include: the testing of drinking water for presence of coliforms to evaluate the safety of water treatment or water delivery systems; monitoring hospital water for the presence of *Legionella;* screening sewage for the presence of polioviruses; or surveilling air in congregate settings for the early detection of airborne biological threats [8, 9].

The fundamental rationale for utilizing environmental surveillance for enteric fever is that contaminated water is critical to its endemicity, and measuring the abundance of typhoidal *Salmonella* in the environment may be an alternative, and potentially more cost efficient, method than clinical surveillance for a disease for which 100 cases per 100 000 population is considered to be a “high burden.” In this paper, we review historical evidence regarding the role of contaminated water in enteric fever transmission, discuss recent studies of *S.* Typhi in the environment, outline methods and challenges for environmental surveillance for enteric fever, and propose concepts for how environmental monitoring could advance enteric fever control.

## Contaminated Water as a Critical Pathway for Typhoidal *Salmonella* Transmission

Although the typhoid bacillus was first identified as the etiologic agent of enteric fever by Karl Eberth in 1880, its transmission through contaminated water was recognized a century earlier by William Budd [10]. In his studies of typhoid outbreaks, Budd observed that “the sewer may be looked upon, in fact, as a direct continuation of the diseased intestine . . . when this fever breaks out in a poor family the discharges from the bowels are thrown, without preparation, either into the common privy, or . . . into the open gutter. From this point, following the line of watershed, this pestilent stuff often makes its way to considerable distances, where, appearing now under the guise of an endemic miasm, which entirely masks its true origin, it may carry the seeds of fresh disease and death into many an unsuspecting household” [11]. Within a decade of the first culture of the typhoid bacillus, the organism was isolated from drinking water in a village experiencing an outbreak in Ireland [12], thereby providing critical evidence for the role of contaminated water in disease transmission. By this time, various ecological evidence identifying a correlation between contaminated water and enteric fever incidence had been generated. In 1884, physician-scientist Henry Baker summarized the situation by stating, “How can typhoid fever be prevented? . . . the reply to the above question may be stated in four words, namely, stop drinking contaminated water” [13].

**Figure 2.**
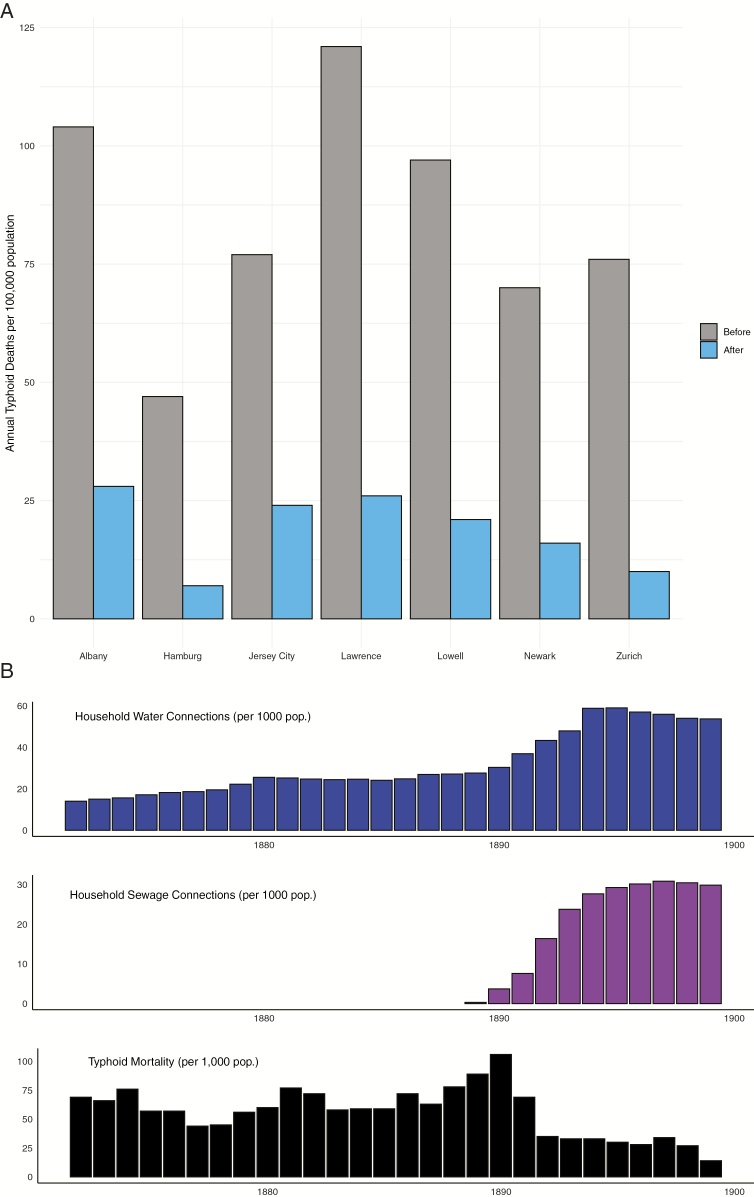
*A*, Average typhoid mortality in US and European cities in the 5 years before and after provision of clean municipal drinking water. *B*, Annual typhoid mortality (bottom) in Buenos Aires, Argentina, following improvements in household water (top) and sewage (middle) connections. Data from Sedgwick and Macnutt [14] and Davison [13].

In the subsequent decades, a substantial reduction in the contamination of drinking water in wealthier countries in North America, Europe, and some locations in Latin America, resulted in a decline in enteric fever incidence. The introduction of municipal water filtration and chlorination in many cities was followed by substantial reductions in typhoid mortality (Figure 2), an effect that was sustained and even accompanied by a decline in mortality from other community acquired infections [14–16]. Cities downstream from those that reduced their typhoid burden through the provision of clean water and sanitation observed an impact on the burden of enteric fever [17]. Through sustained efforts, typhoid was effectively eliminated as a public health problem in most higher income countries; however, it remains a persistent problem in many LMICs, particularly densely populated urban slums, where a continuous clean supply of municipal water is yet to be introduced [2]. In communities where a supply of clean water provision has been provided, subsequent interruption of adequate water treatment has been associated with large-scale enteric fever outbreaks [18, 19], thus highlighting the importance of this transmission route.

The lack of an animal reservoir for *S.* Typhi and *S.* Paratyphi A means that the organisms are transmitted from person to person, either through fecal-oral contact, or via the environment (long cycle transmission) [20]. Localized food- and milk-borne outbreaks of typhoid have been described, but endemic disease is more likely to be sustained by continuous contamination of the water supplies used for household consumption and/or crop irrigation. Crop irrigation was recognized in the 1980s as a driver of typhoid transmission in Santiago, Chile, when it was observed that untreated sewage from the city was being used to water vegetables grown for human consumption [21]. A high incidence of enteric fever caused by *S*. Typhi had perplexed public health officials, given that Santiago was well developed socioeconomically and chlorinated water was serving > 90% of households. The use of Moore swabs for sampling effluent resulted in isolation of *S.* Typhi from irrigation water. The precipitous decline in typhoid incidence after the restriction of using contaminated water for irrigation provided further evidence for this transmission route.

## Challenges in Environmental Surveillance for Typhoidal *Salmonella*

Despite the clear role of contaminated water in the transmission of typhoidal *Salmonella,* monitoring the organisms in the environment to inform enteric fever surveillance techniques has proven challenging. *S.* Typhi is exceptionally difficult to isolate from water and other environmental samples. Although the isolation of *S.* Typhi from contaminated water has been continually reported in the literature over the past 100 years [12, 22–26], the success of this approach has not been consistent, even when fecally contaminated water and sewage samples collected from settings with a high burden of enteric fever have been tested in established laboratories [27]. One of the potential obstacles to isolation of typhoidal *Salmonella* from complex samples is the abundance of other contaminating bacteria, which will invariably outnumber and outgrow *Salmonella.* The use of selective media may overcome a component of this limitation, but recovery of the target organism has been difficult [28, 29].

More consistent success for the detection of *S.* Typhi has been achieved through molecular approaches (eg, real-time polymerase chain reaction [PCR]), even in samples with a high degree of fecal contamination. The key limitations of this approach are the difficulty in confirming a positive result and how to interpret a negative result. Additionally, conventional molecular approaches detect a fragment of genomic DNA and cannot distinguish viable from dead bacteria. Although studies have indicated that typhoidal *Salmonella* may survive for weeks to months in soil or water [28], they ultimately die at varying rates from environmental stresses (or possibly phage lysis [30]), rendering them noninfectious. However, even dead bacteria will test positive by PCR if their DNA avoids degradation. This may not necessarily be a problem if the goal of surveillance is to determine whether typhoidal *Salmonella* are present in an environment, as a general marker of excretion in the population but could hinder inference around transmission. Various molecular methods for assessing bacterial viability have been developed [31], but to our knowledge, none have been successfully applied to typhoidal *Salmonella.*

It is possible that the discrepancies between the ability to culture the organism and the detection of DNA from the pathogen are not due to the aforementioned technical challenges in culture methods nor due to differential viability but rather reflect “differentially culturable” states (sometimes referred to as “viable but nonculturable” states), reflecting relative dormancy of *Salmonella* in aquatic environments. This state has long been described pertaining to nontyphoidal *Salmonella* entering aquatic systems [32], rendering them noninfectious [33], but it has also been characterized in *S.* Typhi [34]. Within a week of entry into groundwater or pond water, a 10-fold difference between viable *Salmonella* cells and culturable cells was consistently identified, and then discrepancy widened over subsequent weeks.

In view of the difficulties with isolating typhoidal *Salmonella* from the environment, it is worth considering whether detecting these organisms is necessary to confirm the risk of enteric fever or whether more general indicators of fecal contamination, such as the presence of coliforms, are sufficient. Even more general characteristics of water and sanitation, such as access to “improved” water sources, have also been suggested as proxies for the risk of enteric fever [35]. However, it is unlikely that physical descriptors of water and sanitation conditions, which have a modest correlation with water quality [35], or even water testing for fecal contamination is sufficient for predicting the local risk of enteric fever. Enteric fever is highly heterogenous spatially and temporally, and recent surveillance studies have found that some communities with extremely poor water and sanitation have a low burden of enteric fever [36–38]. Although it is unlikely that a community with a sufficient sewage treatment system and clean drinking water will have a high incidence of enteric fever, the converse may not be true: some communities with very poor sanitation appear to have a low incidence of typhoid [36]. Environmental testing for typhoidal *Salmonella* may be a more accurate way to classify community risk than proxy indicators of water and sanitation.

In addition to the challenges associated with assays and interpretation of the generated results, there are a number of further issues concerning how to effectively and efficiently deploy these tools in ways that will yield epidemiologically meaningful data on typhoid risk. The general hypothesis pertaining to environmental enteric fever transmission is that *Salmonella*-containing feces are shed into the environment and contaminate water used for human consumption, consequently generating a transmission cycle. The specific ways in which shedding into the environment (eg, open defecation vs open or leaky sewers), contamination of the water supply (eg, mixing with surface water or into water-carrying pipes), and source of water for consumption (eg, drinking water, other household use, or foods contaminated by irrigation water) may impact on the type and location of sample collected. In view of different contamination routes, there are many possible approaches to environmental sampling that may be considered for characterizing potential contamination pathways, depending on the requirement of the surveillance/study. Sampling in proximity to the primary source of consumption, such as drinking water or food, has the advantage of most proximally assessing the risk of infection. In contrast, sampling sewage in the community enables an estimation of the shedding load across a population. Whether measurements of shedding or exposure more accurately reflect disease incidence remains unknown, both are currently being investigated in enteric fever endemic settings.

The broader scientific challenge here is clarifying the relationship between the abundance of typhoidal *Salmonella* in the environment and disease risk. The discrepancy between high frequency of exposure to contaminated water and a relatively low disease incidence was perplexing to early epidemiologists. Editors of *British Medical Journal* commented on the situation in London in 1896: “it must be confessed at first sight there is an apparent absurdity in the statement that a disease which in a year’s time only carries off, as an average, something under 2 persons per 10 000 can result from an infection of a water supply which is common to the whole mass of the population. Still, some large statistical observations have been accumulating during recent years which point strongly in that direction . . . ” [39]. Given the abundance of *S.* Typhi and *S*. Paratyphi A detected in drinking water samples in some endemic cities, it is likely that individuals in these settings frequently ingest the infecting organisms, but only a small minority of these exposures lead to clinical disease. We now understand that infectious dose [40], existing immunity, and genetic background of the exposed individual [41, 42] all may influence the risk of disease after exposure. However, despite these scientific advances, our ability to predict disease risk and population incidence from exposure rates remains limited. Understanding the relationship between the abundance of *Salmonella* in the environment and incidence in the population requires empirical studies from diverse settings, and even then predictions are likely to remain semi-quantitative (eg, “low” vs “high” burden).

## Recent Advances in Our Understanding of the Environment and Typhoid Transmission

Several recent studies have enhanced our understanding of typhoidal *Salmonella* transmission via the environment. Baker and colleagues conducted a comprehensive analysis of S. Typhi and S. Paratyphi A cases diagnosed at a large hospital in the Kathmandu Valley of Nepal, identifying spatial clustering in proximity to municipal water sources and rivers [43]. Genetic clustering of organisms within households and neighborhoods was limited, suggesting that environmental transmission by multiple shedders was dominant. They then undertook testing for typhoidal *Salmonella* in water from 3 communal stone spouts, filtering a liter of water followed by DNA extraction and real-time PCR, which revealed 86% of 118 samples to be positive for *S.* Typhi and 77% positive *S.* Paratyphi A. Despite extensive efforts and use of selective culture media, this group could not isolate typhoidal *Salmonella* from any of the water samples. The group then undertook a systematic study of 10 communal water spouts in the same area, collecting 1.5 liters of water from each site on a weekly basis over a year and testing by real-time PCR [27]. Again, they detected *S.* Typhi and *S.* Paratyphi A at all sites and in a high proportion of samples (*S.* Typhi: 77.5%: *S.* Paratyphi A: 70%). The relative abundance of *S*. Typhi and *S.* Paratyphi A DNA (copies/mL) was the highest during, and immediately following, monsoon rains. Although they isolated a number of pathogenic organisms by culture, including *Vibrio cholerae* 01 and *Shigella dysenteriae* type-1, as well as nontyphoidal *Salmonella* spp., *S.* Typhi or *S*. Paratyphi A were not isolated from any of the samples.

Saha and colleagues evaluated the same method as used in Nepal for molecular testing of water samples from 2 communities in Bangladesh [44]. One site was an urban community in Dhaka with a high incidence of blood culture-confirmed enteric fever, and the other was a rural community in Mirzapur, with a low incidence of enteric fever. In Dhaka, samples were collected from the primary water source of households with a culture-confirmed enteric fever case; among these, 61% of 59 samples tested positive for *S.* Typhi DNA and 24% were positive for *S.* Paratyphi A DNA. In Mirzapur, a geographically representative random sample of water sources was collected, and none of the 33 samples tested contained DNA from either serovar. In northern India, Rani and colleagues applied a molecular-beacon based real-time PCR assay, targeting the same gene (*staG)*, to water samples collected from the Ganga and Yamuna rivers before and after a large religious festival in which millions of devotees bathed in the rivers; *S.* Typhi was detected in surface water and sediments at all 9 sampling sites, and DNA concentrations (copies/mL) were higher in all sites in the month following the festival [45].

Water sampling has also been used reactively amid outbreaks to investigate potential pathways for transmission. In Hyderabad, Pakistan, as part an outbreak investigation for extensively drug-resistant (XDR) *S*. Typhi, Qamar and colleagues utilized a similar set of methods to perform targeted water sampling in communities affected by the outbreak [46]. Among 55 drinking water samples, 12 (22%) were positive for *S.* Typhi DNA. This provided critical evidence to the local government about the function of municipal water in the spread of XDR *S.* Typhi. In a village in rural Pakistan, an outbreak of typhoid with a very high attack rate (300 cases among a population of 500 people) occurred within days of a partial physical cleaning of a well [47]. Among 10 water samples collected from the well, *S.* Typhi was isolated by culture from all 10 (100%), as well as from 65 of 90 (72%) water samples collected from households, underscoring the potential for explosive, point-source outbreaks even in remote areas. *S.* Typhi has been isolated from water amid outbreaks in Nepal and India, though the proportion of positive cultures is typically low, making it difficult to implicate specific events or pathways as causing outbreaks [19, 26]. In other typhoid outbreaks, *S.* Typhi could not be isolated, despite epidemiological evidence of waterborne transmission and the efforts of highly experienced microbiologists [48, 49].

## Towards an Actionable Public Health Approach to Environmental Surveillance for Typhoid

In view of the pivotal role that municipal water plays in the transmission of typhoidal *Salmonella*, and the availability of more sensitive molecular approaches to detecting their presence in water or sewage, there is an opportunity to further develop and validate an approach for environmental surveillance that could provide data to assist in typhoid control in LMICs. There are several mechanisms in which environmental surveillance could be leveraged for control of this disease.

Most urgent is a scalable means of identifying communities with ongoing *S*. Typhi transmission, which should be prioritized for TCV introduction. This step may be particularly important in settings where blood-culture based surveillance data are unavailable or insufficient, and where the presence of *S.* Typhi in drinking water or sewage may be the most easily accessible evidence of pathogen circulation. For this approach to be useful in distinguishing high-risk from low-risk communities, it should be evaluated in settings with diverse incidence and existing high-quality robust surveillance data, in order to characterize the correlation between environmental abundance and disease incidence. It is unlikely that the correlation will be sufficient to make accurate quantitative predictions about incidence, but the goal should be to specify thresholds that could distinguish settings with a high burden from those with low burdens. Several studies are underway with aims of generating such data.

It is important to distinguish this environmental approach for enteric fever surveillance from environmental surveillance programs for poliovirus, which are among the most widely implemented environmental surveillance systems for water-borne pathogens. In poliovirus surveillance, the primary goal is early detection of silently circulating wild-type or vaccine-derived polioviruses, which would trigger an emergency response. As such, the sampling strategy is designed to maximize sensitivity, identifying areas where sewage reflects a large population of individuals, and optimizing the timing and spatial sampling to maximize the chance that a circulating poliovirus would be detected. In contrast, for the goal of characterizing overall population-level transmission, a systematic, representative sampling approach to measure either shedding of (eg, in sewage) or exposure to (eg, in water) *S.* Typhi and S. Paratyphi A is needed. Intentionally sampling high-risk areas, as has been conducted with poliovirus surveillance, undermines the aim of deriving generalized estimates of burden, which are needed to inform vaccine implementation decisions. One approach for achieving representative sampling is to randomly select households within a given community, identify their drinking water source, collect samples, and test for the presence of typhoidal *Salmonella.* Similar approaches could be used for identifying sites for population-representative sewage sampling.

A second potential use of environmental sampling for typhoidal *Salmonella* is to measure, or monitor, the effect of typhoid control interventions. These interventions may include those focused-on water and sanitation, for which measuring the effect on *Salmonella* contamination would a direct and relevant outcome. But such an approach could also be used as a tool for monitoring response to other interventions such as vaccination, to complement other, more traditional, forms of surveillance. Because TCV is unlikely to have an effect on *S.* Paratyphi A (which lacks the Vi polysaccharide), monitoring *S.* Paratyphi A serves as a control outcome to distinguish vaccine-specific effects from other indirect effects on *Salmonella* abundance.

A further potential use for environmental surveillance for enteric fever may be to facilitate disease elimination, whereby surveillance could be used reactively (as is done for poliovirus) to identify the locality of cases or chronic carriers to target further disease control. Because typhoid elimination is most likely to be achieved through sustained improvements in water and sanitation systems in combination with better treatment and vaccine use, we suspect that reactive surveillance for identifying and treating asymptomatic shedding is unlikely to be necessary in communities once they achieve sufficient progress in the provision of clean water. Notably, in countries that have previously eliminated typhoid as a public health problem, reactive programs for case identification have not played a major role.

Finally, the evidence of typhoidal *Salmonella* in municipally supplied drinking water may serve a critical function in advancing advocacy for addressing the water and sanitation crises facing much of the developing world. The provision of uninterrupted, clean drinking water in LMICs will not be achieved without substantial political will and corresponding financial investment. Although the widespread presence of fecal coliform bacteria in the municipal water supply of major cities in Africa and South Asia is well known [50], these indicator bacteria have not yet motivated policy makers to make major commitments. It is possible that the knowledge that government provided water is delivering *S*. Typhi, a pathogen associated with widespread disease and death, and increasingly resistant to effective drugs, may increase the urgency of policy makers to provide definitive solutions [51].

Scaling up environmental surveillance for typhoidal *Salmonella* in resource-constrained settings will require the development of standardized tools, validation alongside population-based surveillance, training of personnel, laboratory infrastructure, and resources to support and sustain their use. Although real-time PCR-based methods appear most promising at this time, many settings currently lack the capacity to perform these assays. The costs and complexity of operationalizing prospective, blood culture-based surveillance are also substantial, if not greater, and yet some form of typhoid-specific surveillance data are needed. Countries that are conducting poliovirus environmental surveillance may have an opportunity to leverage resources for typhoid environmental surveillance, particularly as polio control activities are being phased out in many countries.

A number of low-cost alternative approaches to conventional lab-based DNA extraction and real-time PCR are under development, including portable and “lab-on-a-chip” approaches, but none have yet been field tested for *S.* Typhi or S. Paratyphi A detection. Novel, low-cost approaches to *S.* Typhi and S. Paratyphi A detection in water would expedite their scalability in resource-constrained settings. In parallel, efforts should continue toward identifying more effective culture-based methods that overcome the current challenges of sensitivity, as this has potential to contain costs while providing valuable information, including phenotypic antimicrobial resistance and genotypes, that are not available through current PCR-based approaches.

## CONCLUSION

The advent of sensitive molecular techniques for detection of typhoidal *Salmonella* in water and sewage provides a potential alternative to blood culture-based clinical surveillance for characterizing community burden of typhoid. Although the laboratory tools have been successfully deployed in proof-of-concept studies in typhoid-endemic settings, the major challenge ahead is in determining how to effectively and efficiently utilize them to characterize transmission routes and community burden of typhoid. This will require testing various sampling approaches in ecologically diverse communities with population-based clinical surveillance as a reference standard to enable their interpretation in settings with unknown disease burden. If successful, environmental surveillance could play an important role in generating actionable data to inform the public health response to typhoid.

## Notes

***Financial support*.** This work was supported by the Bill and Melinda Gates Foundation (INV-000572; OPP1113007) and the Center for Innovation in Global Health at Stanford University.

***Supplement sponsorship.*** This supplement is funded with support from the Coalition against Typhoid Secretariat, housed at the Sabin Vaccine Institute in Washington, DC and made possible by a grant from the Bill & Melinda Gates Foundation.

***Potential conflicts of interest***. J. A. receives grant support from Bill and Melinda Gates Foundation and Center for Innovation in Global Health. S. S. receives grant support from Bill and Melinda Gates Foundation, National Institutes of Health and Research, World Health Organization, Pfizer, GlaxoSmithKline, and Sanofi Pasteur. S. L. receives grant support from Bill and Melinda Gates Foundation. All other authors report no potential conflicts. All authors have submitted the ICMJE Form for Disclosure of Potential Conflicts of Interest. Conflicts that the editors consider relevant to the content of the manuscript have been disclosed.
